# Symptom Dimensions of the Psychotic Symptom Rating Scales in Psychosis: A Multisite Study

**DOI:** 10.1093/schbul/sbu014

**Published:** 2014-06-13

**Authors:** Todd S. Woodward, Kwanghee Jung, Heungsun Hwang, John Yin, Laura Taylor, Mahesh Menon, Emmanuelle Peters, Elizabeth Kuipers, Flavie Waters, Tania Lecomte, Iris E. Sommer, Kirstin Daalman, Remko van Lutterveld, Daniela Hubl, Jochen Kindler, Philipp Homan, Johanna C. Badcock, Saruchi Chhabra, Matteo Cella, Sarah Keedy, Paul Allen, Andrea Mechelli, Antonio Preti, Sara Siddi, David Erickson

**Affiliations:** ^1^Department of Psychiatry, University of British Columbia, Vancouver, British Columbia, Canada;; ^2^BC Mental Health and Addictions Research Institute, Vancouver, British Columbia, Canada;; ^3^Department of Pediatrics, University of Texas Health Science Center, Houston, TX;; ^4^Department of Psychology, McGill University, Montreal, Québec, Canada;; ^5^Department of Psychology, and the BRC of the South London and Maudsley NHS Foundation Trust, Institute of Psychiatry, King’s College London, London, UK;; ^6^North Metro Health Service Mental Health, and School of Psychiatry and Clinical Neuroscience, University of Western Australia, Perth, Australia;; ^7^Department of Psychology, University of Montreal, Montreal, Québec, Canada;; ^8^Department of Psychiatry, Universitair Medisch Centrum, Utrecht, The Netherlands;; ^9^Department of Psychiatric Neurophysiology, University Hospital of Psychiatry, University of Bern, Bern, Switzerland;; ^10^School of Psychology, University of Western Australia, Crawley, Western Australia, Australia;; ^11^Department of Psychiatry and Behavioral Neuroscience, University of Chicago, Chicago, IL;; ^12^Department of Psychosis Studies, King’s College London, London, UK;; ^13^Department of Education, Psychology, Philosophy, University of Cagliari, Cagliari, Italy;; ^14^Fraser North Early Psychosis Program, Royal Columbian Hospital, New Westminster, British Columbia, Canada

**Keywords:** psychosis, schizophrenia, hallucinations, delusions, symptom rating scales, structural equation modeling, principal component analysis

## Abstract

The Psychotic Symptom Rating Scales (PSYRATS) is an instrument designed to quantify the severity of delusions and hallucinations and is typically used in research studies and clinical settings focusing on people with psychosis and schizophrenia. It is comprised of the auditory hallucinations (AHS) and delusions subscales (DS), but these subscales do not necessarily reflect the psychological constructs causing intercorrelation between clusters of scale items. Identification of these constructs is important in some clinical and research contexts because item clustering may be caused by underlying etiological processes of interest. Previous attempts to identify these constructs have produced conflicting results. In this study, we compiled PSYRATS data from 12 sites in 7 countries, comprising 711 participants for AHS and 520 for DS. We compared previously proposed and novel models of underlying constructs using structural equation modeling. For the AHS, a novel 4-dimensional model provided the best fit, with latent variables labeled *Distress* (negative content, distress, and control), *Frequency* (frequency, duration, and disruption), *Attribution* (location and origin of voices), and *Loudness* (loudness item only). For the DS, a 2-dimensional solution was confirmed, with latent variables labeled *Distress* (amount/intensity) and *Frequency* (preoccupation, conviction, and disruption). The within-AHS and within-DS dimension intercorrelations were higher than those between subscales, with the exception of the AHS and DS Distress dimensions, which produced a correlation that approached the range of the within-scale correlations. Recommendations are provided for integrating these underlying constructs into research and clinical applications of the PSYRATS.

## Introduction

The Psychotic Symptom Rating Scales (PSYRATS) is an instrument developed for quantification of the multidimensional features of the psychotic symptoms of hallucinations and delusions.^1^ It is often used in research settings focusing on people with schizophrenia and psychosis to gather additional information regarding hallucinations and delusions over and above that provided by a general symptom rating interview, such as the Positive and Negative Syndrome Scale (PANSS)^2^ or the Scale for the Assessment of Positive Symptoms (SAPS).^3^ Increasingly, it has been used as an outcome measure in trials of psychological therapy^4,5^ and for comparing individuals with psychotic experiences with and without a “need for care.”^6–8^ Some groups have also used the PSYRATS for quantifying hallucinations and delusions in nonclinical samples although the PSYRATS items may not match their experiences particularly well.^9^ A number of other instruments are available for assessment of specific aspects of voices, such as the beliefs about and response to voices,^10^ power appraisals,^11^ interpretation,^12^ acceptance,^13^ mindfulness,^14^ and relationship with voices,^15^ but do not include phenomenological/topographical aspects of voices such as frequency, loudness, and location, and therefore may complement the PSYRATS but would not replace it.

The PSYRATS is comprised of 17 items inquiring about the specific dimensions of hallucinations and delusions, with each item being rated from 0 (absent) to 4 (severe). The PSYRATS has 2 subscales: the auditory hallucinations subscale (AHS) consisting of 11 items, and the delusions subscale (DS) consisting of 6 items. The AHS items are Frequency, Duration, Location, Loudness, Origin, Negativity (Amount/Degree), Distress (Amount/Intensity), Disruption, and Controllability. The DS items are Preoccupation (Amount/Duration), Conviction, Distress (Amount/Intensity), and Disruption. Although the AHS and DS subscales have face validity, they do not necessarily reflect the psychological constructs underlying the scale. Identification of these constructs is important for many clinical and research contexts (eg, measuring change in cognitive/biological processes), because when certain PSYRATS items cluster together, that clustering may be caused by some underlying etiological process (eg, duration and disruption items on the AHS could all be affected by sustained hyperactivity in brain networks involving speech-related and auditory perception regions).

In several studies, patterns of intercorrelation between individual PSYRATS items have been analyzed in an exploratory fashion, and some encouraging consistencies have been observed. For example, for both the AHS and the DS, frequency-of-experience related items (eg, voices and/or thoughts are continuous) separated from distress-related items in all studies.^1,16–18^ This suggests that separable phenomenological and etiological processes underlie duration and distress for both hallucinations and delusions. In other words, although duration and distress may share some underlying etiological processes (ie, the dimensions may be correlated), they are measurably distinct in some way.^1,16–18^

Despite these consistencies in the published studies on the dimensional structure of the PSYRATS, a number of inconsistencies have also emerged. For example, the number of dimensions underlying the AHS have been proposed to be 3 (emotional, physical, and cognitive) in some studies (Drake et al^16^, *N* = 257; Haddock^1^, *N* = 71) but 4 (emotional, physical, control, and cognitive) in others (Kronmuller et al^17^, *N* = 200; Steel et al^18^, *N* = 276). The major difference between the 3- and 4-dimensional solutions was that the 4-dimensional solution placed disruption/control and location/origin onto distinct dimensions (referred to as “control characteristics” and “cognitive interpretation,” respectively), whereas they were merged in the 3-dimensional solution (under “cognitive interpretation”). With respect to the DS, all studies agreed on 2 underlying dimensions although disruption was placed with amount/duration of delusions in some studies ^1^,^16^,^17^ but with distress in another.^18^

These inconsistencies may have been caused by analysis of site-specific groups of patients because some samples were derived from research studies with specific inclusion and exclusion criteria.^16,18^ This may adversely affect generalizability to all patients with psychosis, as would other factors such as site-specific rater-training methods. To resolve these inconsistencies, a multisite data set is helpful because this serves to increase external validity and generalizability. In order to compare the competing models directly, confirmatory dimension reduction can be used. In this study, we compared the proposed dimensional structures of the PSYRATS using structural equation modeling (SEM) in an international multisite sample of patients with psychosis and schizophrenia and explored associations of each dimension with demographic variables. This work was undertaken as part of the activities of the 1st and 2nd International Consortium on Hallucination Research conferences in London and Durham, in 2011 and 2013.^19,20^

## Methods

### Participants

The numbers of participants in the final sample, along with total scores for the AHS and DS, are listed in table 1, columns 3–6. The group of Sommer et al (Utrecht) is a University Medical Center, in which patients are interviewed for both clinical and research purposes. The Waters site (Perth) is a clinical research center attached to the University of Western Australia. The Badcock/Chhabra site (Perth) is a clinical research center; participants were tested as part of a doctoral research thesis (S.C.). The Bern group on hallucination research (Hubl/Kindler/Homan) is a University Hospital with inpatient and outpatient treatment facilities, where all patients are assessed with the PSYRATS, and some participate in diverse research studies (eg, neuroimaging). The Allen/Mechelli (London) site recruited ultrahigh-risk for psychosis individuals from a specialist prodromal clinic (Outreach and support in South London), and the Peters/Kuipers site (London) is a specialist psychological therapy service (Psychological Interventions Clinic for outpatients with Psychosis), both in the South London and Maudsley National Health Services Foundation Trust. The Lecomte site (Montreal) recruits individuals with early psychosis from 2 first-episode programs who were participating in a study on group cognitive behavioral therapy (CBT) for psychosis. The Erickson site (Vancouver) is an early psychosis program and the participants were enrolled in a study investigating the efficacy of CBT. The Woodward site (Vancouver) is a research setting whereby all patients are participating in research studies on cognition and/or functional neuroimaging. Cella (Swansea) recruited participants from outpatient community mental health services as part of a research project. The Siddi/Preti site (Cagliari) is a psychiatric service, where participants were enrolled in a study investigating the neuropsychological correlates of verbal auditory hallucinations. The Keedy site (Chicago) is a research laboratory in a university medical center setting whereby patients are participating in research studies on neurocognition with naturalistic observation of treatment changes.

**Table 1.** Sample Size, Mean Age, Mean Duration of Illness, and Mean Subscale Score as a Function of Site

Site	Testing Location	AHS (*N*)	Age	DI	AHS Total	DS (*N*)	Age	DI	DS Total	AHS + DS (*N*)	Age	DI	AHS + DS Total
1	Utrecht	208	35.76	15.63	29.72	n/a	n/a	n/a	n/a	n/a	n/a	n/a	n/a
2	Perth	32	36.38	13.94	24.63	n/a	n/a	n/a	n/a	n/a	n/a	n/a	n/a
3	Perth	32	40.63	18.13	26.59	n/a	n/a	n/a	n/a	n/a	n/a	n/a	n/a
4	Bern	54	39.57	13.04	24.46	54	39.57	13.04	8.81	54	39.57	13.04	33.28
5	London	43	24.35	0.51	12.19	46	24.41	0.61	9.57	42	24.45	0.61	22.00
6	Montreal	31	23.71	3.41	11.48	29	26.93	3.97	8.38	26	26.69	3.97	17.62
7	Vancouver	15	23.80	2.50	15.40	16	24.06	2.41	8.94	15	23.80	2.41	24.93
8	London	219	39.92	n/a	26.91	280	39.53	n/a	15.04	146	39.38	n/a	41.71
9	Vancouver	10	37.90	17.10	28.10	61	31.79	12.99	12.40	8	37.25	12.99	38.44
10	Swansea	34	37.74	15.91	17.21	34	37.74	15.91	7.97	34	37.74	15.91	25.18
11	Cagliari	18	37.67	10.56	25.61	n/a	n/a	n/a	n/a	n/a	n/a	n/a	n/a
12	Chicago	15	42.87	20.07	26.80	n/a	n/a	n/a	n/a	n/a	n/a	n/a	n/a
*N*s		711	711	407	711	520	520	230	520	325	325	170	325

*Note*: Site: 1. Daalman/Sommer/van Lutterveld; 2. Waters/Badcock/Maybery; 3. Badcock/Chhabra; 4. Hubl/Kindler/Homan; 5. Allen/Mechelli; 6. Lecomte; 7. Erickson; 8. Peters/Kuipers; 9. Woodward; 10. Cella; 11. Siddi/Preti; 12. Keedy. AHS = Auditory Hallucinations Scale; DS = Delusions Scale. n/a = not available. Total score is computed by summing all items on the respective scales. DI = Duration of Illness.

The AHS data consisted of 711 patients who responded to all 11 items of the hallucination scale. Demographic variables (and the *N*s on which they are based) were as follows: the mean age was 36.54 (*SD* = 10.86; *N* = 711), mean illness duration was 12.55 years (*SD* = 10.72; *N* = 407), and mean years of education was 11.89 (*SD* = 3.13; *N* = 392). The sample was 58.4% male (*N* = 711), and 91.4% were right handed (*N* = 394). Schizophrenia spectrum diagnoses were as follows, as specified by the diagnostic manuals (ie, DSM-IV or ICD-10) or according the procedures of the individual site: schizophrenia: 48.4%; schizoaffective: 5.8%; psychosis NOS: 9.0%; first-episode psychosis: 3.7%; at-risk mental state: 2.4%; and seeking treatment for psychosis: 30.8%.

The DS data consisted of 520 patients who responded to all 6 items of the delusions scale. Demographic variables (and the *N*s on which they are based) were as follows: the mean age was 36.39 (*SD* = 10.5; *N* = 520), mean illness duration was 9.05 years (*SD* = 8.85; *N* = 230), and mean years of education was 12.22 (*SD* = 3.33; *N* = 213). The sample was 60.6% male (*N* = 520), and 93.8% were right handed (*N* = 161). Diagnosis information was as follows: schizophrenia: 31.5%; schizoaffective: 3.8%; psychosis NOS: 1.9%; first-episode psychosis: 5.0%; at-risk mental state: 3.8%; and seeking treatment for psychosis: 53.8%.

The AHS + DS data consisted of 325 patients who responded to both the 11 items of the AHS and 6 items of the DS scale. Demographic variables (and the *N*s on which they are based) were as follows: the mean age was 35.52 (*SD* = 10.63; *N* = 325), mean illness duration was 8.48 (*SD* = 9.01; *N* = 170), and mean years of education was 11.85 (*SD* = 3.48; *N* = 153). The sample was 59.4% male (*N* = 325), and 97.1% were right handed (*N* = 104). Diagnosis information was as follows: schizophrenia: 34.5%; schizoaffective: 5.5%; psychosis NOS: 2.2%; first-episode psychosis: 8.0%; at-risk mental state: 4.9%; seeking treatment for psychosis: 44.9%.

Sample size, age, duration of illness, and mean subscale score are presented as a function of site in table 1 columns 3–6 for AHS, columns 7–10 for DS, and columns 11–14 for AHS + DS.

### Data Analysis Strategy

#### Structural Equation Modeling.

For SEM, we used Generalized Structured Component Analysis (GSCA).^21^ GSCA is a component-based method of SEM, which approximates a latent variable as a component or weighted composite of observed variables (as in principal component analysis [PCA]). Thus, GSCA allows direct computation of latent variable scores.^22^ Latent variable scores provide one value per participant, specifying the strength of each symptom dimension for that participant. Latent variable scores, as with PCA, can be used to associate symptom dimensions with demographic variables such as gender and length of illness.^23,24^ Using GSCA, individual latent variable scores can be directly and uniquely computed and are optimized to explain the maximum proportion of variance in PSYRATS items. Unlike with GSCA, in factor-based SEM,^25,26^ a latent variable is approximated as a common factor. Accordingly, individual latent variable scores are not unique, such that different scores can lead to the same model fit, known as factor score indeterminacy.^27^ GSCA latent variable scores are computed by postmultiplying the corresponding original item scores by weight estimates.^21^ These individual scores can then be related to demographic variables using standard analysis procedures.^24^

In addition to direct computation of latent variable scores, component-based SEM has 2 additional advantages over factor-based SEM. First, it is more flexible with respect to model specification, and accordingly, GSCA almost never encounters problems of nonconvergence, or convergence to improper solutions (eg, negative variance estimates), a condition frequently affecting covariance-based methods.^27^ Second, GSCA does not rely on stringent distributional assumptions, such as the multivariate normality of observed variables, an assumption that is likely violated in PSYRATS data due to some items consisting of a disproportionate number of low scores. Thus, GSCA is considered beneficial due to direct computation of latent variable scores, and application to complex models and/or data sets with high potential for multivariate nonnormality. For these reasons, GSCA was used to compute latent variable scores optimized to explain the maximum proportion of variance in PSYRATS items according to the investigated models.

## Hallucinations Scale

### Models

Several investigations have explored the dimensional structure of the AHS using exploratory factor analysis.^1,16–18^ These studies suggested either a 3-dimensional model, consisting of emotional characteristics, physical characteristics, and cognitive interpretation,^1,16^ or a 4-dimensional model consisting of emotional characteristics, physical characteristics, control characteristics, and cognitive interpretation.^17,18^ In this study, we evaluated how well these models fit the data, and a third (novel) model was also developed using exploratory PCA. In order to use exploratory PCA to develop a novel model, care must be taken to avoid circular reasoning. Specifically, if exploratory PCA were carried out on the full data set and GSCA FIT values for that model were compared to competing models from the literature on the same data set, the exploratory PCA-derived model would provide the best fit because it was derived from the same data on which it was tested. To avoid this circularity, split-half methodology was used. This involves randomly splitting the data set into 2 halves, deriving the exploratory PCA model on the first half of the data but comparing the fit of the exploratory model to that of the competing models from the literature on the preserved half of the data. For this study, if the novel exploratory PCA model produced FIT values on the preserved half of the data that were superior to those from past work, this model could then be considered preferable to the other models.

To evaluate the models statistically, overall goodness-of-fit measures, named FIT in GSCA, were compared, with higher relative FIT values indicating superior model fit.^21^ FIT values can be interpreted as the percentage of variance in the PSYRATS items accounted for by the model.^21^ Differences between FIT statistics for competing models were tested for significance using bootstrapping.^28^ Specifically, we fitted 2 models to each of *n* bootstrap samples (*n* = 100 in this study), which were randomly drawn from the original data set with replacement, and calculated the differences of FIT values computed on the 2 models. Following this, we applied a 1-sample *t*-test to the difference scores, using degrees of freedom equal to *n* = 1 (99 in this case). If the mean of the difference scores was not statistically different from zero, this would indicate that there is no reliable mean difference in FIT between the 2 models. This methodology can result in significant (and reliable) differences between FIT values that differ by only a few percentage points. Thus, differences in these FIT statistics cannot be interpreted in the same fashion as differences between correlations coefficients computed on one sample,^29^ which typically require a substantial gap in values to be considered reliably different. As for all SEM fit statistics, it is currently not known how differences in FIT statistics translate into practical differences in subscale computation applications and effect sizes. In this study, we simply conclude that model A would be preferred over model B if the FIT value for model A is reliably (ie, significantly) higher than that for model B, with the real-world impact of that FIT difference being currently unknown.

### Results

The loadings for the 3- and 4-dimensional models estimated by GSCA are provided in table 2. In both, all items loaded significantly onto their respective latent variables, indicating that the latent variables were well constructed. GSCA indicated that the FIT value of the 3-dimensional model was 0.520, such that 52% of the total variance of all observed PSYRATS items was accounted for by the specified latent variables. FIT for the 4-dimensional model was 0.531, such that 53.1% of the total variance of all observed PSYRATS items was accounted for by the specified latent variables. The bootstrapped *t*-test between the 3- and 4-dimensional models was significant, *t*(99) = 5.90, *P* < .001, indicating that there is a reliable mean difference in FIT between the 2 models, justifying choice of the 4-dimensional model over the 3-dimensional model.

**Table 2.** Auditory Hallucinations Scale Model Loadings

	Item	3-Dimension Model FIT = 0.520		4-Dimension Model FIT = 0.531		Exploratory Model FIT = 0.541	
		Label	Loading	Label	Loading	Label	Loading
6	Negative content amount	EMO	0.91	EMO	0.91	EMO	0.89
7	Negative content degree		0.87		0.87		0.85
8	Distress amount		0.91		0.91		0.90
9	Distress intensity		0.88		0.88		0.88
1	Frequency	PHY	0.83	PHY	0.90	PHY	0.88
2	Duration		0.88		0.90		0.84
4	Loudness		0.71	CON	0.68	LDN	1.00
10	Disruption	COG	0.74		0.80	PHY	0.82
11	Control		0.77		0.81	EMO	0.68
3	Location		0.69	COG	0.83	COG	0.83
5	(Beliefs regarding) origin		0.73		0.84		0.84

*Note*: EMO, motional, PHY, physical, COG, cognitive; CON, control; LDN, loudness. All loadings were significant at *P* < .05. FIT value for full AHS sample is reported (*N* = 711).

We also tested the fit of a novel model based on exploration of the current data set. We randomly split the data into 2 halves and carried out a pseudo-exploratory PCA on 1 half (whereby the analysis was restricted to 4 components, as was suggested by model fits reported above). This resulted in a novel (4-dimensional) model that was characterized by the Cognitive (COG) dimension from the original 4-dimensional model, the Physical (PHY) dimension from the original 4-dimensional model with Disruption added, and the Emotional (EMO) dimension from the 3- and 4-dimensional models with the Control item added. Loudness (LDN) remained independent from all other dimensions and was derived from the Control/Loudness (CON) dimension from the original 4-dimensional model (Disruption and Control moved to PHY and EMO, respectively, thereby dismantling the CON dimension). We then compared the 3- and 4-dimensional models with the new exploratory model on the preserved half of the data set. For this analysis, we found that, as with the full data set, the 3-dimensional model provided the poorest FIT (0.522), and the 4-dimensional and new exploratory models provided substantially better FIT (0.530 and 0.543, respectively). To statistically compare the FIT values of these 3 models using bootstrapping, a 1-way ANOVA was carried out with 1 between-subjects factor with 3 levels (the 3 models), and this was highly significant, *F*(2, 297) = 72.83, *P* < .001. This was followed up by a Scheffe’s test to examine which models were different from one another. The results of the multiple comparisons test suggested that, as above, the 4-dimensional model FIT was superior to the 3-dimensional model (mean difference = −0.0064; 95% *CI* = −0.0107, *P* < .005) and that the new exploratory model FIT was superior to the 3- and 4-dimensional model (mean difference from the 4-dimensional model = −0.0142; 95% *CI* = −0.0185, *P* < .001).

## Delusions Scale

### Models

Previous investigations into the dimensional structure of the DS using exploratory factor analysis suggested 2-dimensional models with different configurations of items, labeled COG and EMO.^1,16–18^ One model placed Disruption with Amount/Duration,^1,16,17^ and the contrasting model placed Disruption with Distress.^18^ These 2 models are contrasted here using SEM. An exploratory PCA analysis on the full data set did not produce a novel model over and above these 2, so the split-half methodology used with the AHS reported above was not necessary.

### Results

The loadings for the disruption-with-duration and disruption-with-distress models estimated by GSCA are provided in table 3. In both models, all items loaded significantly onto their respective latent variables. GSCA indicated a FIT value of 0.563 for the disruption-with-duration model and 0.558 for the disruption-with-distress model. The bootstrapped *t*-test comparing the models was significant, *t*(99) = 5.06, *P* < .001, indicating that disruption-with-duration could be chosen over disruption-with-distress.

**Table 3.** Delusion Scale Model Loadings

	Item	Disruption-With-Distress FIT = 0.558		Disruption-With-Duration FIT = 0.563	
		Dimension	Loading	Dimension	Loading
1	Preoccupation amount	COG	0.87	COG	0.85
2	Preoccupation duration		0.90		0.88
3	Conviction		0.82		0.80
4	Disruption	EMO	0.75		0.75
5	Distress amount		0.90	EMO	0.95
6	Distress Intensity		0.92		0.95

*Note*: COG, cognitive; EMO, emotional. All loadings were significant at *P* < .05.

## Hallucinations and Delusions Scales

### Models

In the previous set of analyses, we concluded that a novel 4-dimensional model was optimal for AHS and that the 2-dimensional disruption-with-duration model was optimal for DS. We now investigate the dimensional structure of PSYRATS as a whole, ie, a 6-dimensional model of PSYRATS based on the subsamples of the AHS and DS, who completed both scales. We did not use the split-half exploratory analyses methodology on these data because the sample size of the AHS + DS data set was the smallest of the 3. We instead preserved the optimal dimension structure derived from the full AHS and DS samples.

### Results

The loadings for the 6-dimensional model of AHS and DS are provided in table 4, along with Intraclass Correlation Coefficients (ICC), which provide estimates of reliability for each subscale.^30^ All items loaded significantly onto their respective latent variables, and GSCA indicated that the FIT value was 0.558.

**Table 4.** Full 6-Dimensional Model Loadings

Old Label	New Label	ICC	Item		Loading
H-EMO	H-DIS	.93	6	Negative content amount	0.89
			7	Negative content degree	0.85
			8	Distress amount	0.90
			9	Distress intensity	0.88
			11	Control	0.68
H-PHY	H-FRQ	.87	1	Frequency	0.88
			2	Duration	0.84
			10	Disruption	0.82
H-COG	H-ATT	.67	3	Location	0.83
			5	(Beliefs regarding) origin	0.84
H-LDN	H-LDN	n/a	4	Loudness	1.00
D-EMO	D-DIS	.93	4	Distress amount	0.95
			5	Distress intensity	0.95
D-COG	D-FRQ	.87	1	Preoccupation amount	0.85
			2	Preoccupation duration	0.88
			3	Conviction	0.80
			6	Disruption	0.75

*Note*: H- denotes auditory hallucination scale and D- denotes delusion scale. DIS, distress; FRQ, frequency; ATT, attribution; LDN, loudness; ICC, intraclass correlation; n/a, not applicable. Coefficient (ICC) estimate of reliability. All loadings were significant at *P* < .05, FIT = 0.558.

#### Labeling Dimensions.

In order to better reflect the parallels between the AHS and DS dimensions and to sharpen the concepts conveyed by the dimensional structure, we generated new labels for the dimensions. The H-EMO and D-EMO items were commonly renamed Distress (H-DIS and D-DIS, respectively), to highlight the fact that although emotion-related symptoms (ie, negative content) are not captured by the DS, distress-related symptoms are, making the AHS and DS parallel with respect to distress but not emotion. The H-PHY and D-COG dimensions were commonly renamed Frequency (H-FRQ and D-FRQ, respectively) to emphasize that frequency-of-occurrence items formed the basis of both dimensions. Notably, both the H-FRQ and the D-FRQ scales included the Disruption item. The H-COG dimension was renamed Attribution (H-ATT) to emphasize the involvement of items indexing the internal/external attribution of location and source. H-LDN remained unchanged.

#### Correlations Among Dimensions.

The correlations among the 6 latent variables are presented in table 5 and indicate that the AHS dimensions and the DS dimensions were highly correlated within each scale but that the correlations between scales were reduced, with the H-DIS and D-DIS dimensions producing the highest cross-scale correlation. Statistical comparison of these correlations^31^ demonstrated that the highest cross-scale correlation (0.47 between the H-DIS and D-DIS dimensions) did not differ significantly from the lowest within-scale correlation (0.55 between H-FRQ and H-LDN), suggesting that this 1 cross-scale correlation involving distress was not reliably different from the lower range of the within-scale correlations. In contrast, the lowest within-scale correlation (0.55 between H-FRQ and H-LDN) was significantly higher than all the remaining cross-scale correlations (ie, those other than the H-DIS and D-DIS correlation discussed above), ranging in magnitude from 0.21 to 0.38 (all *P*s < .005).

**Table 5.** The Coefficients Obtained From the 6-Dimensional Model

	H-DIS	H-FRQ	H-ATT	H-LDN	D-DIS	D-FRQ
H-DIS	1.00					
H-FRQ	0.77	1.00				
H-ATT	0.66	0.66	1.00			
H-LDN	0.61	0.55	0.58	1.00		
D-DIS	0.47	0.31	0.36	0.28	1.00	
D-FRQ	0.37	0.32	0.38	0.21	0.76	1.00

*Note*: H- denotes auditory hallucination scale and D- denotes delusion scale. DIS, distress; FRQ, frequency; LDN, loudness; ATT, attribution. All correlation coefficients were significant at *P* < .05.

#### Relationships to Demographic Variables.

Correlations of latent variable scores with available demographic variables were computed using the largest available samples. Age was not included in this analysis because it is highly confounded with length of illness (and site), and diagnosis was not included due to the wide range of labels used across sites (eg, first episode, psychosis NOS, at-risk mental state, and seeking treatment for psychosis). Computed on the AHS full sample (*N* = 711), H-DIS and H-LDN significantly differed between males and females, *t*(709) = 2.91, *P* < .005, *t*(709) = 4.78, *P* < .001, respectively, as females (*M* = .13, *M* = .21, respectively) had higher latent variables scores than males (*M* = −.09, *M* = −.15, respectively). For the DS full sample (*N* = 520), D-DIS significantly differed between males and females *t*(518) = 2.64, *P* < .01, with females (*M* =.14) having higher latent variables scores than males (*M* = -.09). These results suggested that women report more distress in their hallucinations and delusions, as well as louder hallucinations, relative to men.

There was also evidence that scores on all aspects of hallucinations, but not delusions, increased with length of illness. Computed on the AHS full sample (*N* = 407 with length of illness information), all latent variables were significantly and positively correlated with length of illness (*r* = .20, .19, .19 and .25 with H-DIS, H-FRQ, H-ATT, and H-LDN, respectively, all *P*s < .05). No correlations were significant for the DS latent variable scores. However, length of illness (and the related age variable) was strongly confounded with site, and AHS total score differed between sites (see table 1). Therefore, conclusions regarding length of illness must be considered highly tentative because they may also be attributed to age and/or site. Parallel association analyses computed on the smaller AHS + DS sample (*N* = 325) gave essentially identical results, so are not reported here. There were no significant associations between the latent variable scores and handedness or education.

## Discussion

In this study, PSYRATS models previously identified in the literature, and a novel model derived from the current data, were empirically compared using SEM. For the AHS, a 4-dimensional model provided the best fit, with dimensions labeled DIS (Distress/Negative Content/Control), FRQ (Frequency/Duration/Disruption), ATT (Attribution of voices: Location and Origin), and LDN (Loudness item only). For the DS, a 2-dimensional solution was confirmed, with DIS (Distress) and FRQ (Preoccupation, Conviction, and Disruption) emerging. A model including both the AHS and DS dimensions showed higher latent variable score correlations within the AHS and DS scales than between, with the exception of the H-DIS and D-DIS dimensions, which produced a between-scale correlation that could be considered within the range of the high within-scale correlations. This provides the most definitive analyses of the factor structure of the PSYRATS to date because it involves the largest sample size, has the most representative patients (across research and clinical settings and across countries), and makes an empirical comparison of competing models using confirmatory methods (viz., SEM).

The fact that these sets of PSYRATS items cluster together can lead to identification of underlying etiological processes. For example, the preoccupation, conviction and disruption items on the DS could all be affected by a cognitive bias such as a bias against disconfirmatory evidence^32^ or jumping to conclusions bias (JTC),^33,34^ contributing to the formation of the D-FRQ dimension. Similarly, the frequency, duration, and disruption items on the AHS could all be affected by sustained hyperactivity in brain networks involving speech-related and auditory perception regions,^35,36^ contributing to the formation of the H-FRQ dimension. For practical application of these results (eg, computation of an outcome measure), summation of items can be used in place of computation of latent variable scores. The recommended procedure for all dimensions is to sum the specific items comprising each of the H-DIS, H-FRQ, H-ATT, H-LDN (only one item), D-FRQ, and D-DIS dimensions (as listed in table 4). For example, the H-FRQ dimension would be computed by simply summing the Frequency, Duration, Disruption items on the AHS.

The differentiation of separate constructs within the AHS and DS may be important for the study of etiological processes involved in psychosis, such as change in symptoms and their underlying brain networks in response to treatment. Treatment approaches may reasonably target certain dimensions, such as distress,^18^ and these dimension may relate to cognitive biases underlying delusions in different ways. For example, 1 study using the 3-dimensional solution found that COG and EMO were related to cognitive biases in healthy and clinical samples of people experiencing hallucinations, but PHY was not.^7^ In another study,^37^ different dimensions of delusions changed at different rates over time in response to medication, and the JTC bias predicted treatment response on some dimensions but not others. In another article in this special issue,^38^ it is argued that CBT for psychosis trials have not used optimal outcome measures to assess change because CBT aims to reduce distress and disruption and not change physical characteristics of voices. Thus, the dimensions likely to change with a given intervention may vary, and the primary outcome measure should be specified depending on the goal of the study. However, it should be noted that some CBT for psychosis protocols specifically target disruption due to voices but not their frequency.^39,40^ In this case, the use of individual items on the PSYRATS may be more sensitive to change than using the H-FRQ dimension, which combines frequency and disruption. Dimension reduction methods such as PCA and GSCA focus on the common variance between test items, but the item specific variance may also be important to capture under some conditions.

The current results also suggest that treatments reducing frequency and duration of voices would be most likely to also reduce disruption of daily life (H-FRQ includes Frequency, Duration and Disruption) and that voices with negative content (eg, threatening voices) are the most distressing and difficult to control (H-DIS includes Negative Content, Distress, and Control). Accordingly, research has shown that negative content can distinguish clinical voice-hearers from nonclinical ones (see another article in this special issue).^8^ However, it should be noted that these results do not imply that negative content can be equated to distress,^41^ only that negative content and distress tend to co-occur and that these items would be expected to correlate in data samples.

The H-ATT dimension may be of particular interest, as it includes the (Beliefs About) Origin item, and the variance that it shares with Location. Beliefs and experiences regarding the originating source of the voices appears to tap into the personal meaning attributed to these experiences more than any other PSYRATS item. This refers to a common frame of reference for voice-hearing experiences, mainly internal (cognitive/biological explanation) or external (mystical or divine explanation), a key concept to the Hearing Voices Movement (HVM), which is discussed in another article in this special issue.^42^ The HVM suggests that an effective practice for supporting distressed individuals with hallucinations should involve trying to understand such a frame of reference, and supporting a change in the person’s relationship with their voices with an exploration of who/what the voices represent. The reframing of appraisals is also a key focus for many psychological therapies, which hold that a change in the personal meaning assigned to the voice can lead to better coping and reduced intensity/distress.^43,44^

GSCA was selected for this analysis partly because it allows direct computation of latent variable scores, allowing observation of intercorrelations of the AHS and DS scales. This led to the observation that the within-AHS-scale and within-DS-scale dimension correlations were higher than those between, with the exception of the hallucinations and delusions dimensions indexing distress (H-DIS and D-DIS), which produced a correlation not reliably different from the range of the within-scale correlations. This correlation may be attributable to the distressing aspects of highly negative voices leading to distressing (eg, paranoid) delusions. Correlation of latent variable scores with demographic variables led to the observation that the hallucination dimension indexing distress and emotional content (H-DIS) was higher in women than men and that hallucinations were rated as louder in women relative to men. Although it has previously been reported that hallucinations (and not delusions) are more common in women than men,^45^ to our knowledge, this is the first study reporting sex differences in severity of specific aspects of hallucinatinos. Finally, scores on all aspects of hallucinations, but not delusions, increased with length of illness although this effect was highly confounded with age and site, whereby some sites focused on patients in the very early stages of their illness (see table 1). The confounding nature of age, length of illness, and site in this sample made the length of illness finding very difficult to interpret and therefore this result must be considered highly tentative.

An important caveat of this work is that it is not known whether the identified dimensions exhaustively represent all relevant dimensions of hallucinations and delusions because these were derived from PSYRATS items, which were selected based on interviews with hallucinating patients.^1(p881)^ Also, other more general scales (eg, PANSS) were not readily available for investigations of PSYRATS external validity because a range of general scales were used at the different sites.

The PSYRATS provides quantification of phenomenological/topographical aspects of voices, such as frequency, loudness, and location, that are not available with other scales and also provides a more detailed assessment of delusions than is typically available with more general scales. Our results suggest that clinical and research use of the PSYRATS (eg, to evaluate symptom change) could benefit from computation of the 6 subscales listed in table 4, involving summing the specific items comprising each of the dimensions. The loudness item would simply be left as an individual item. This methodology could potentially allow clinicians and researchers to discriminate finer aspects of change in hallucinations or delusions and provides methodology for identifying more homogenous subgroups of participants with auditory hallucinations or delusions. As mentioned above, this can aid in the search for underlying etiological processes. Future studies may investigate how the dimensions of the PSYRATS are associated with other more general scales such as the PANSS^46^ or SAPS,^3^ or with other specific aspects of psychotic symptoms, such as omnipotence of beliefs about hallucinations,^10^ or mindful response.^47^

## Funding

Canadian Institutes of Health Research and the Michael Smith Foundation for Health Research (to T.S.W.); British Columbia Schizophrenia Society Foundation and Mitacs Elevate (to K.J.); United States National Institute of Mental Health award (K23MH092702 to S.K.); Regione Sardegna, Italy (PRR-MAB-A2011-19251 to S.S.).
